# Engineering hematopoietic stem and progenitor cells to generate red blood cells as viral traps against HIV-1

**DOI:** 10.1016/j.omta.2026.201799

**Published:** 2026-06-29

**Authors:** Sofia E. Luna, William N. Feist, Kaya Ben-Efraim, Alex Pendergast, Freja K. Ekman, Britney Hernandez-Naughton, Megan Merritt-Garza, Kathleen Romero, Zak Kostamo, Nelson A. Amorin, Nicole M. Johnston, Hana Y. Ghanim, Vivien Sheehan, Amanda M. Dudek, Matthew H. Porteus

**Affiliations:** 1 Institute for Stem Cell Biology & Regenerative Medicine, Stanford University School of Medicine, Stanford, CA 94305, USA; 2 Department of Pediatrics, Stanford University School of Medicine, Stanford, CA 94305, USA; 3 Aflac Cancer Center and Blood Disorders, Department of Pediatrics, Emory University School of Medicine, Atlanta, GA, USA; 4 Department of Genetics, Stanford University School of Medicine, Stanford, CA 94305, USA; 5 Department of Microbiology and Immunology, Carver College of Medicine, University of Iowa, Iowa City, IA 52242, USA

**Keywords:** hematopoietic stem cell, hematopoietic stem cell transplant, genome-editing, CRISPR/Cas9, homology directed repair, CD4, HIV-1, viral trap, red blood cell

## Abstract

Canonical HIV-1 entry into target cells depends on binding to CD4 as a primary receptor. Because of this, use of the CD4 receptor as a viral trap (a decoy receptor used to prevent infection of target cells) is a promising strategy for the treatment of HIV-1. One challenge in using CD4 viral traps is maintaining enough of the decoy receptor in circulation to remain effective. Here, we present a strategy to produce cell-based CD4 viral traps by engineering hematopoietic stem and progenitor cells (HSPCs) to express the CD4 receptor in red blood cell (RBC) progeny. This takes advantage of the ability of the HSPC to repopulate the blood system for a lifetime, while leveraging the fact that RBCs greatly outnumber any cell targeted for infection. CRISPR-Cas9-engineered HSPCs efficiently express CD4 on their cell surface after differentiation to the RBC lineage *in vitro*. Fusion of CD4 to glycophorin A (GPA) and introduction of a truncated erythropoietin receptor (tEPOR) leads to increased CD4 expression and enrichment of edited cells (CD4-GPA-tEPOR) to levels capable of neutralizing HIV-1 pseudovirus *in vitro*. In sum, this work presents a potential strategy for the one-time delivery of CD4-RBC viral traps through autologous transplantation of engineered HSPCs.

## Graphical abstract

**Figure:**
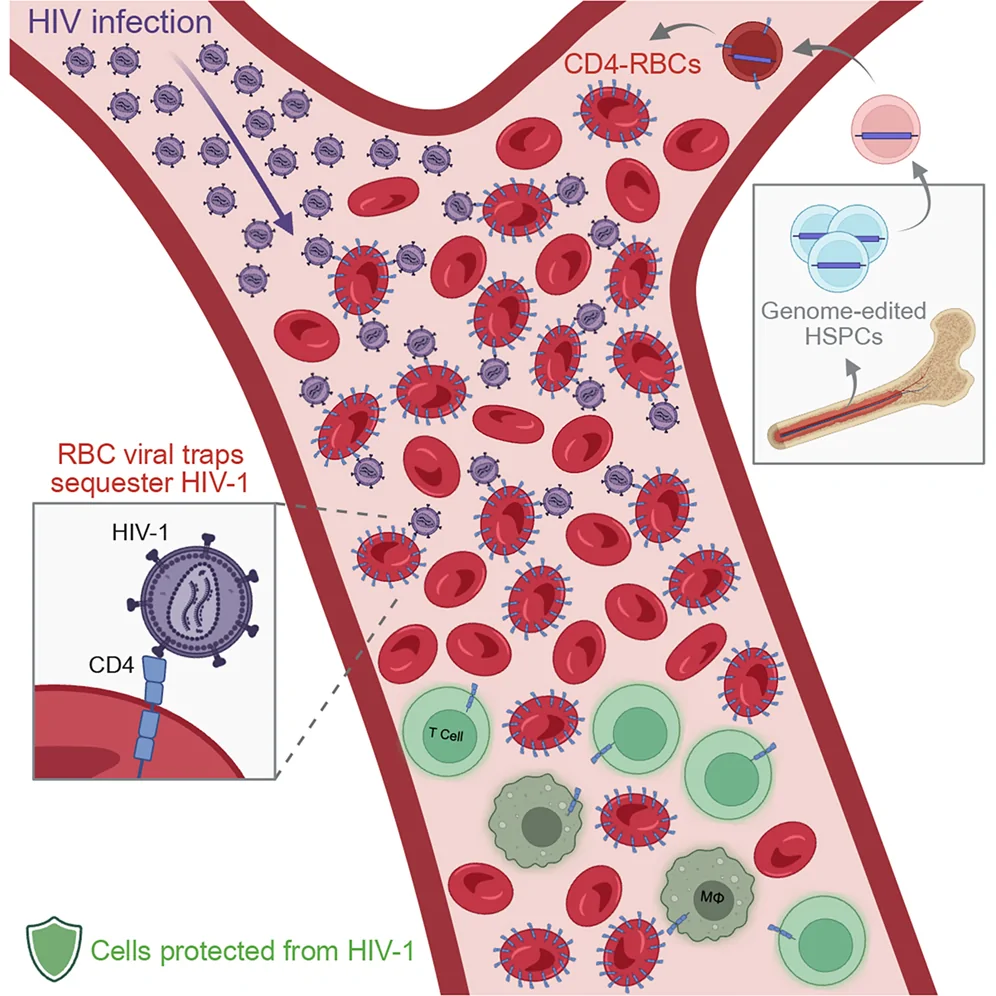

## Teaser

Porteus and colleagues demonstrate that human blood stem cells can be engineered such that the derived red blood cells express high levels of CD4 providing an HIV trap. This potentially durable suppressive therapy for HIV might obviate the need for patients to take anti-retroviral therapy for the rest of their lives.

## Introduction

A key step in viral infection is binding to cell surface receptors that facilitate entry into host cells. Consequently, blocking receptor binding has been a primary focus in the development of new antiviral therapies, which has resulted in the creation of new antibodies, peptides, and small molecules that inhibit receptor and/or co-receptor binding.^1^^,^^2^ Another strategy to block viral entry is the use of viral traps, which employ “decoy” receptors that engage with viral particles to prevent binding of target cells. Red blood cells (RBCs) modified to carry decoy receptors represent an intriguing option for the development of viral traps. There are roughly 25 trillion RBCs in adult circulation,^3^ making them the most abundant cell type in the body, vastly outnumbering any other cell type targeted for infection. Moreover, mature RBCs are enucleated and lack the necessary machinery for viral replication, eliminating the potential for viral propagation within RBCs, and are naturally cleared from the body by the reticuloendothelial system.^4^^,^^5^ Importantly, human RBCs have been reported to function naturally as viral traps against adenovirus type 5 (Ad5), and are hypothesized to have evolved in humans to protect against infection.^6^ Thus, the development of custom RBC viral traps engineered to express decoy receptors builds upon this naturally occurring viral resistance strategy.

One obstacle in utilizing RBCs as viral traps is the difficulty in engineering the enucleated cells to express the receptor of interest. Non-genomic methods of modification have been reported, including adsorption of nanoparticles^7^ and targeting proteins via encapsulation,^8^ antibodies,^9^ and sortase-mediated reactions.^10^ However, these methods are limited by the short survival of the modified RBCs in circulation (maximum reported ∼28 days) due both to dissociation of the modifications and natural clearance of the cells.^10^ An alternative strategy is genetic engineering of erythroid progenitor cells and subsequent differentiation into enucleated RBCs.^11^ This approach offers a permanent alteration of engineered RBCs, yet transfused RBCs only persist for around 50–60 days^12^ and therefore require repeated administration to combat chronic infections. To overcome these obstacles, we seek to leverage the long-term engraftment potential of hematopoietic stem and progenitor cells (HSPCs) as a continual source of RBCs and their progeny. Here, we present a strategy to genetically engineer HSPCs using targeted integration into an erythroid specific locus for erythroid-specific expression of cell surface receptors for long-term production of RBC viral traps against HIV-1.

HIV-1 infects cells by binding to the primary receptor, CD4, leading to a conformational change that allows for binding to one of two co-receptors, CCR5 or CXCR4, followed by fusion with the host cell membrane.^13^ Although co-receptor switching and drug resistance mutations are common, all strains of HIV-1 rely on binding to the CD4 receptor for canonical infection. Consequently, viral mutations to escape binding will also lead to an accompanying decrease in viral fitness.^14^^,^^15^ Thus, prior work has sought to capitalize on this reliance by repurposing exogenous CD4 as a decoy to prevent binding to target cells (primarily CD4^+^ T cells and macrophages). Early works employed delivery of a soluble form of CD4 (sCD4), which successfully neutralized laboratory strains of HIV-1 but showed limited efficacy in a clinical trial.^16^ An improvement on this strategy involved combining CD4 with an immunoglobulin Fc region (CD4-Ig)^17^ and a CCR5-mimetic sulfopeptide (eCD4-Ig),^18^ of which the latter protected rhesus macaques against simian/human immunodeficiency virus (SHIV) challenges.^18^^,^^19^ A recent strategy employed CD4-expressing virus like particles (VLPs),^15^ and, later, RBCs,^11^ which allowed for formation of CD4 receptor clusters on the VLP or RBC surface. These clusters mimic natural high avidity binding seen on cell surfaces, thereby mitigating the potential for viral escape.^11^^,^^15^ Using a cell-based carrier to deliver CD4 has the potential to dramatically improve the half-life of delivered drugs,^5^ yet these cells will eventually require re-administration. Despite great advancement in the efficacy of CD4 mimetic compounds, they all require repeated dosing to remain effective due to natural clearance from circulation. In contrast, transplantation of engineered HSPCs presents a one-time strategy for lifelong production of RBC viral traps.

In this study, we engineer HSPCs so that, upon reconstitution of the hematopoietic system, they will generate CD4-expressing RBCs that serve as viral traps against HIV-1. Key to our strategy is precisely editing HSPCs for erythroid-specific expression, so that only derived RBCs and no other blood cell lineages errantly express CD4. This lineage specific expression harnesses the critical properties of RBCs to serve as highly abundant viral traps, while leaving other lineages unperturbed. We demonstrate efficient genetic engineering of HSPCs that successfully differentiate into CD4-expressing RBCs *in vitro*. We also introduce an RBC-specific selective advantage in the edited cells through introduction of a truncated erythropoietin receptor (tEPOR)^20^ and show that derived RBCs significantly reduce viral infection *in vitro*. Taken together, we introduce a strategy for inhibition of chronic viral infection through autologous transplantation of HSPCs engineered for RBC-specific expression of viral receptors.

## Results

### Precision integration of CD4 expression cassettes in human HSPCs

**Figure 1:**
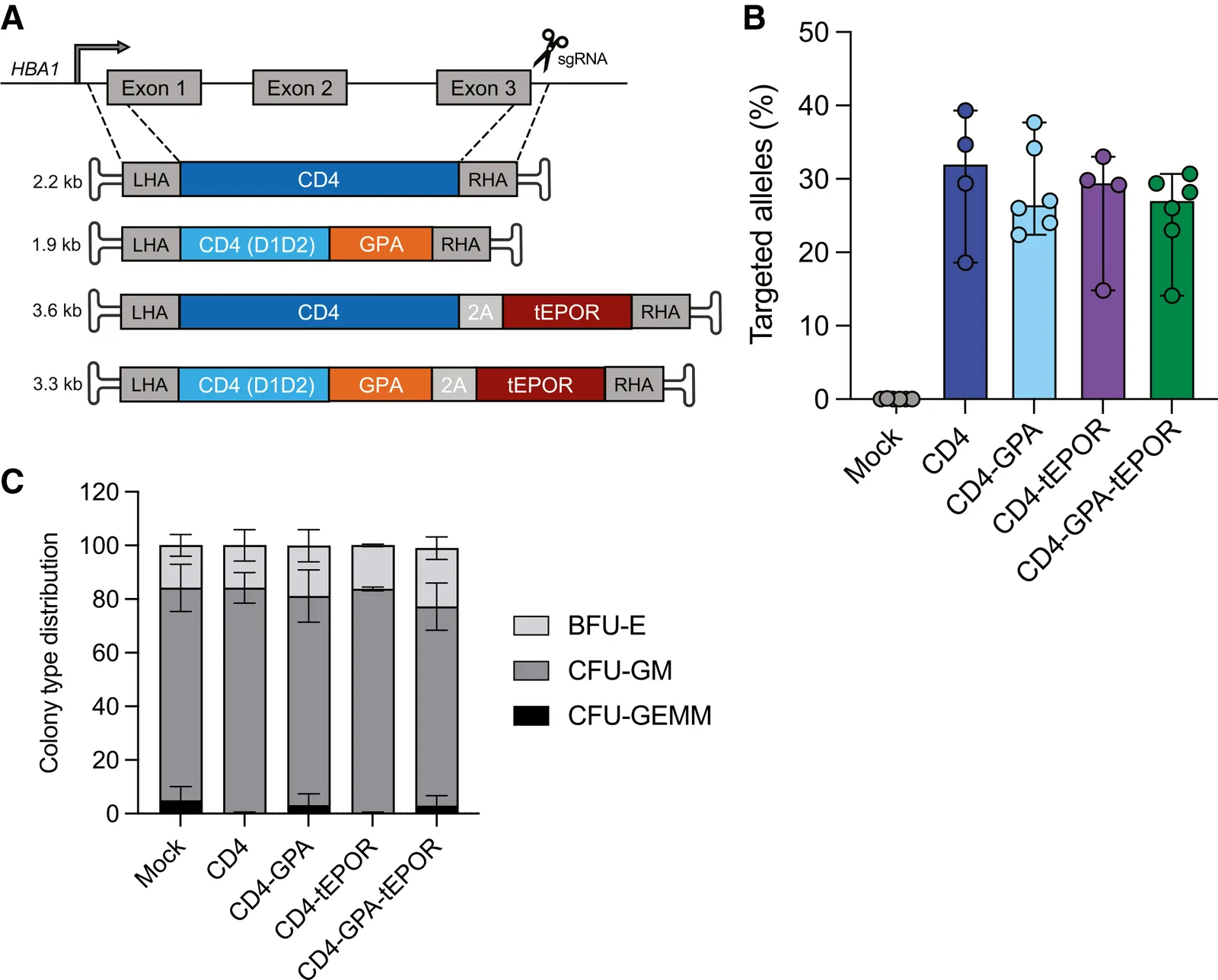
Efficient engineering of CD4 receptor cassettes at the *HBA1* locus (A) Schematic of genomic editing strategy at the *HBA1* locus for targeted integration of CD4 expression cassettes delivered by AAV6. (B) Percentage of alleles with targeted integration for each CD4-expression cassette measured 2–3 days post-editing. Each AAV6 was used at 2500 vector genomes/cell. *n* = 6 independent HSPC donors for mock, CD4-GPA, and CD4-GPA-tEPOR, *n* = 4 for CD4 and CD4-tEPOR. Bars represent median ± 95% confidence interval. (C) CFU assay showing mock edited cells versus cells edited with each CD4 expression cassette. Bars represent relative frequency of each progenitor colony type: CFU-GEMM, multi-potential granulocyte, erythroid, macrophage, megakaryocyte progenitor cells; CFU-GM, colony forming unit-granulocytes and monocytes; BFU-E, erythroid burst-forming units; bars represent mean ± SD. *n* = 5 biological donors for mock, CD4-GPA, CD4-GPA-tEPOR, *n* = 3 biological donors for CD4 and CD4-tEPOR.

Recent studies have shown that CD4 decoys, such as CD4-expressing RBCs and eCD4-Ig, can be used to efficiently neutralize HIV infection.^11^^,^^15^^,^^18^^,^^19^^,^^21^ Based on this concept, we hypothesized that knock-in of a CD4 expression cassette by homology-directed repair (HDR) into the *HBA1* locus of HSPCs would allow for high levels of lineage-specific production of CD4-expressing RBC progeny to act as viral traps, while minimizing the potential of CD4 expression in other blood cell types. When we analyze the expression of *HBA1* in the Human Protein Atlas database, we find it is only expressed in RBCs and has a mean expression level of >50,000 normalized transcripts per million (nTPM), among the highest expressed genes in any cell type.^22^^,^^23^ Targeting *HBA1* functions as a safe harbor integration due to the natural redundancy of *HBA1* with *HBA2* and the fact that individuals with bi-allelic inactivation of *HBA1* have the benign condition of α-thalassemia trait. While the random integration of recombinant AAV is extremely rare because it encodes no rep gene and Rep is not packaged into the vector, the design of the construct without a promoter also limits any potential for toxicity from an off-target integration event. This serves as an advantage over some therapies which have led to insertional oncogenesis by retroviral and lentiviral vectors, as seen in gene therapy trials for SCID-X1,^24^ chronic granulomatous disease,^25^ and X-linked adrenoleukodystrophy.^26^ We therefore used ribonucleoprotein (RNP)-based CRISPR-Cas9 editing along with recombinant AAV6 delivered donor templates to target CD4 expression cassettes into the *HBA1* locus by whole gene replacement.^27^ We employed a previously described single-guide RNA (sgRNA) that specifically cuts in the 3′ untranslated region (UTR) of *HBA1* while leaving *HBA2* intact. Previous work has shown that this sgRNA cuts with high specificity with no measurable off-target activity.^27^ We then designed donor templates with homology arms flanking the coding sequence of *HBA1* (Figure 1A).^27^ Based on prior work by Hoffman et al. (2021), who used semi-random lentiviral integration of CD4,^11^ we tested two forms of CD4 donor templates: the entire CD4 coding sequence (codon optimized and hereafter termed “CD4”) and a CD4-GPA fusion protein, which physically links the codon-optimized extracellular D1D2 region of human CD4 to the N terminus of glycophorin A (GPA) (hereafter termed “CD4-GPA”). This earlier study found that codon-optimization of CD4 leads to increased expression of the receptor, and that use of CD4-GPA increased CD4 expression on erythrocytes since GPA is naturally highly expressed on the surface of RBCs.^11^ Further, previous work has shown use of tEPOR to increase erythroid proliferation in an erythropoietin-dependent manner and enrich for tEPOR-edited cells over the course of erythroid differentiation.^20^^,^^28^ We therefore designed two additional vectors containing a tEPOR following a 2A self-cleavage peptide on the 3′ end of either CD4 or CD4-GPA (Figure 1A) to enhance erythropoietic output of gene-edited cells.

To test targeted integration frequency of the four CD4 donor cassettes, we delivered high fidelity Cas9 protein complexed with the *HBA1* sgRNA into HSPCs via electroporation, followed by immediate transduction with individual AAV6 donors. Two to three days post-editing, we analyzed the targeted integration frequency at *HBA1* by droplet digital PCR (ddPCR). After optimization of AAV6 vector dose (Figure S1A), we achieved an average allelic integration frequency of approximately 30% using 2500 vector genomes/cell (vg/cell) (Figure 1B). We also verified that insertions and deletions (indels) were being created at the *HBA1* target site to facilitate targeted integration, though it is important to note that these indels are in the 3′UTR and unlikely to ablate protein expression (Figure S1B).^27^

To assess the impact of the editing on the differentiation capacity of HSPCs, we performed a colony forming unit (CFU) assay and found that consistent with prior reports of RNP/AAV6-mediated editing, there was a decrease in total colony formation compared to mock-edited cells (Figure S1C).^29^^,^^30^^,^^31^^,^^32^^,^^33^ Additionally, there was a reduced number of CFU-GEMM colonies in the CD4 and CD4-tEPOR conditions, while the lineage distribution of colonies was not affected in the CD4-GPA and CD4-GPA-tEPOR conditions, indicating that targeted integration of the CD4-GPA expression cassettes does not impede myelo-erythroid differentiation potential of HSPCs *in vitro* (Figure 1C). Single-cell colonies were also genotyped, and we found most integration events were mono-allelic, with a much smaller proportion of colonies demonstrating bi-allelic editing (Figure S1D).

### Edited HSPCs differentiate into CD4-expressing RBCs *in vitro*

**Figure 2:**
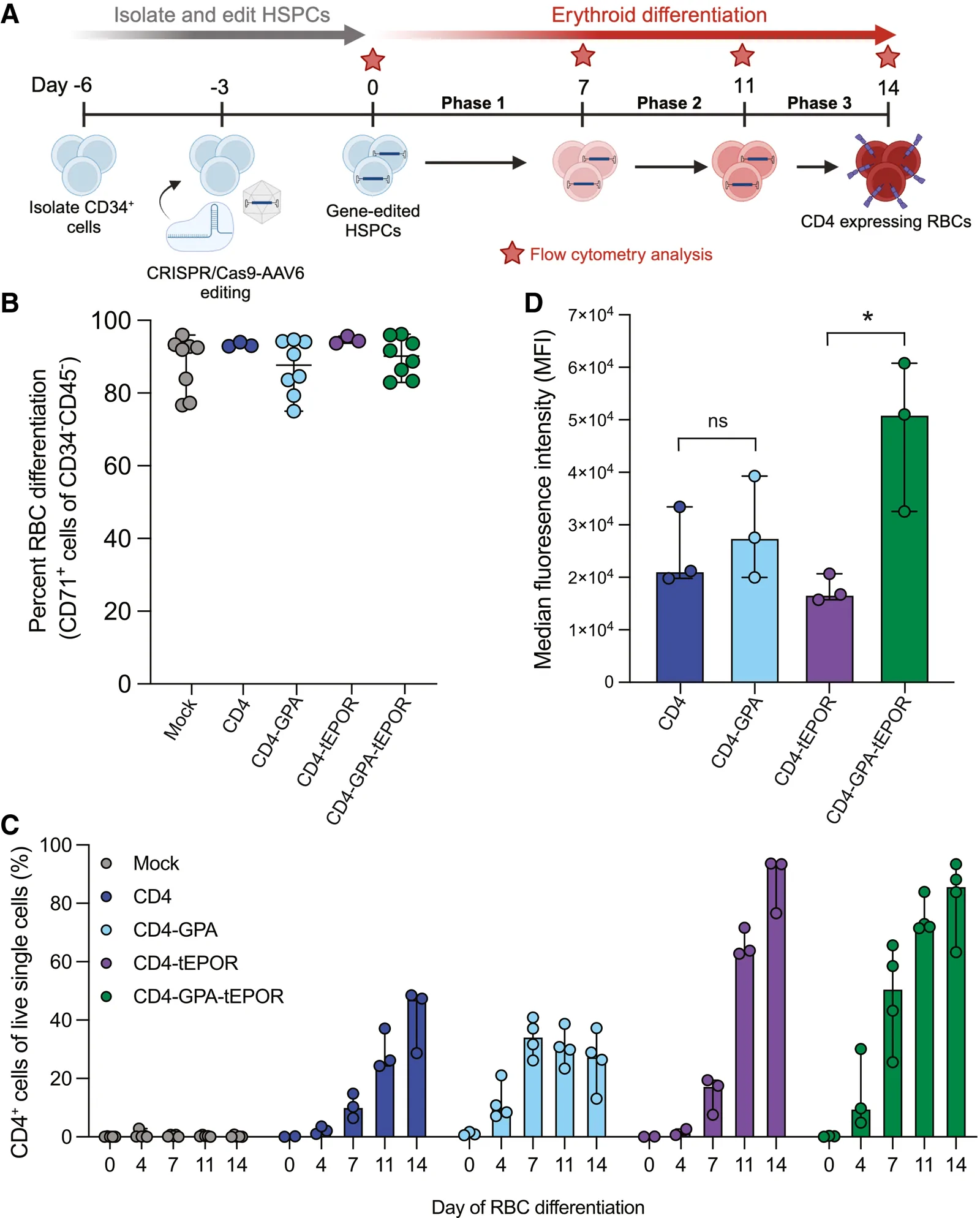
Genome-edited HSPCs show robust expression of CD4 following erythroid differentiation (A) Diagram showing workflow of genome-editing and subsequent RBC differentiation of HSPCs. (B) Percentage of CD71^+^ cells of CD34^-^CD45^-^ cells on day 14 of RBC differentiation. *n* = 8 for mock, CD4-GPA, CD4-GPA-tEPOR, *n* = 3 for CD4 and CD4-tEPOR. (C) Percentage of CD4^+^ cells of live single cells on each day of RBC differentiation. *n* = 6 for mock, *n* = 4 CD4-GPA, CD4-GPA-tEPOR, *n* = 3 on day 4–14 for CD4 and CD4-tEPOR on day 4–14 and *n* = 2 on day 0. (D) Median fluorescence intensity (MFI) of CD4 signal within CD4^+^ cells on day 14 of RBC differentiation. *n* = 3 for all conditions. ns, *p* = 0.5919 for CD4 vs. CD4-GPA, ^∗^*p* = 0.0224 for CD4-tEPOR vs. CD4-GPA-tEPOR by unpaired two-tailed *t* test. All bars represent median ± 95% confidence interval and all replicates represent independent HSPC donors.

Next, the HSPCs targeted with each CD4 expression cassette were differentiated into RBCs using a previously described 14-day *in vitro* erythroid differentiation protocol (Figure 2A).^34^ On day 14, we assessed erythroid differentiation by flow cytometry using established RBC markers (CD34^−^CD45^−^CD71^+^). We observed no appreciable differences in phenotypic RBC differentiation markers between mock edited and CD4-edited cells. On average >85% of cells in all conditions expressed CD71 on day 14, indicating that addition of CD4-expression cassettes does not impede RBC differentiation (Figure 2B). Of note, GPA, a commonly used RBC cell surface marker, was omitted during quantification of RBC differentiation due to antibody cross-reactivity with the GPA expressed in some of the editing conditions. However, we found no differences in GPA expression in the CD4 and CD4-tEPOR edited conditions compared to mock-edited cells (Figure S2A).

After confirming there was no defect in differentiation, we quantified relative CD4 receptor expression on the surface of cells throughout differentiation in each editing condition. As expected, because CD4 expression is driven by the RBC-specific endogenous *HBA1* promoter, there was no detectable CD4 expression in any of the conditions at the beginning of differentiation (termed day 0). By the end of RBC differentiation (day 14), an average of 85% of cells expressed CD4 in the CD4-tEPOR and CD4-GPA-tEPOR conditions, while an average of 33% of cells displayed CD4 in the constructs lacking tEPOR (Figure 2C). As we hypothesized, by expressing the protein from the robust *HBA1* promoter in the RBC lineage, we found that CD4 was highly expressed on the cell surface at day 14 (Figure S2B). Additionally, we found that CD4 expression was indeed RBC-specific and only detectable in cells expressing the erythroid marker CD71 (Figure S2C). These data highlight that our knock-in expression constructs show high levels of CD4 expression, are highly lineage specific, and the total number of CD4^+^ cells is augmented by the inclusion of tEPOR.

Allelic integration frequencies of the CD4 expression cassettes at the *HBA1* locus were monitored by ddPCR throughout differentiation and confirmed that targeted integration frequencies correlate with CD4 cell surface expression (Figure S3A). As previously shown in other tEPOR-based expression systems,^20^ CD4-tEPOR and CD4-GPA-tEPOR-edited cells enriched over the course of differentiation, achieving an average targeted integration frequency of almost 70% on day 14. This represented an average fold increase of 2.8× for CD4-tEPOR and 2.9× CD4-GPA-tEPOR from day 0 to day 14 of differentiation. The knock-in rate of non-tEPOR constructs remained at around 30% throughout differentiation (Figure S3A). We also found as expected that indels at *HBA1* were maintained at day 14 of differentiation in the CD4 and CD4-GPA conditions and proportionately decreased in the tEPOR conditions corresponding to an increase in knock-in cells throughout differentiation (Figure S3B). Because we observed robust enrichment over time from an already high knock-in frequency, we then investigated how strong the enrichment phenotype could be under lower efficiency editing conditions. We edited HSPCs with half the AAV6 vector dose (1,250 vg/cell) using the CD4-tEPOR and CD4-GPA-tEPOR cassettes to evaluate the extent to which tEPOR-edited cells could enrich over the course of RBC differentiation. While initial editing frequencies were indeed lower at day 0 (average 18.5% for CD4-tEPOR and 17.2% CD4-GPA-tEPOR), edited cells enriched further throughout differentiation (average fold increase of 3.8× for CD4-tEPOR and 3.5× CD4-GPA-tEPOR from day 0 to day 14), reaching similar integration frequencies to those observed in the cells edited with the higher AAV6 dosage by the end of RBC differentiation (Figure S3C). We also validated that our genome-editing and RBC differentiation workflow could be applied to peripheral blood derived CD34^+^ HSPCs and found similar trends in CD4 expression levels and RBC differentiation outcomes (Figures S4A–S4C).

Lastly, we examined the abundance of CD4 receptors on the cell surface by measuring the median fluorescence intensity (MFI) of CD4 signal by flow cytometry. We found both CD4-GPA and CD4-GPA-tEPOR constructs exhibited higher levels of CD4 receptor expression when compared to the CD4 and CD4-tEPOR constructs, respectively, with CD4-GPA-tEPOR yielding the highest MFI (Figure 2D). We then compared the relative expression level of CD4 on the CD4-GPA and CD4-GPA-tEPOR RBCs to stimulated and unstimulated CD4^+^ T cells and found that the CD4-expressing RBCs had 2 to 10-fold higher expression, measured by MFI, of CD4 on the cell surface (Figure S3D). Based on these results, both the greater total cell number (∼50:1 RBC to T cell ratio) and higher CD4 expression levels suggest that free virions would have a substantially higher likelihood of engaging CD4 on the surface of engineered RBCs than on T cells, demonstrating their strong potential to act as viral traps.

### CD4-GPA expressing myelo-erythroid cell line neutralizes HIV-1

**Figure 3:**
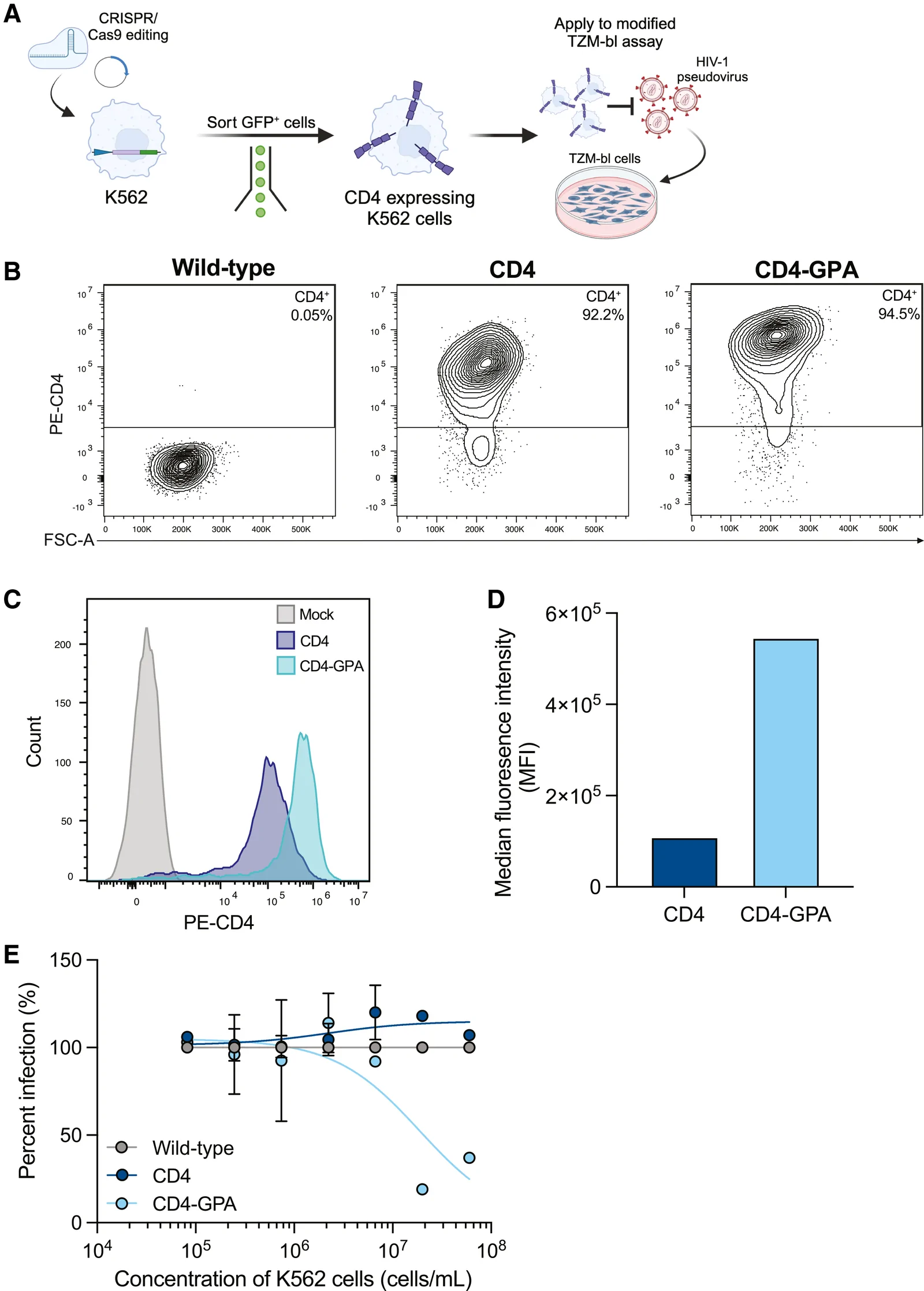
Stable cell line expressing CD4 neutralizes HIV-1 pseudovirus (A) Schematic of workflow for K562 stable cell line generation and testing. (B) Flow cytometry plots of CD4 expression on wild-type K562s and K562s engineered with CD4 expression cassettes driven by the Ef1α promoter after sorting and expansion of GFP^+^ cells. (C) Histogram showing relative CD4 expression of wild-type, CD4, and CD4-GPA K562s. (D) Median fluorescence intensity (MFI) of CD4 signal within CD4^+^ cells for CD4 versus CD4-GPA K562s. *n* = 1. (E) Inhibition of infection with TRO11 HIV-1 pseudovirus using wild type, CD4, or CD4-GPA engineered K562s (*n* = 1, data points and error bars represent mean ± standard deviation of technical duplicate infections, some duplicates were excluded due to mechanical error).

To investigate whether the cells edited with the full-length CD4 cassette or CD4-GPA fusion cassette have superior neutralization capacity, we tested the potential of cells expressing each construct to neutralize HIV-1 pseudovirus using a CD4 or CD4-GPA expressing K562 (myelo-erythroid) stable cell line. The cell lines were created by targeting cassettes carrying each receptor (CD4 or CD4-GPA) driven by a strong ubiquitous promoter, Ef1α, and co-expressed with green fluorescent protein (GFP) to the *CCR5* safe-harbor locus (Figure 3A). RNP targeting *CCR5*^35^ was nucleofected into K562 cells along with a CD4-2A-GFP or CD4-GPA-2A-GFP plasmid. We then used fluorescence-activated cell sorting (FACS) to isolate a pure population of GFP^+^ cells. Post-sorting, we confirmed that both CD4 and CD4-GPA K562 cell lines contained >90% CD4^+^ cells (Figure 3B). We found that the CD4-GPA expressing cells exhibited much higher levels of expression of the CD4 receptor on the cell surface, with an MFI almost 5 times higher than its CD4 counterpart (Figures 3C and 3D). To test the ability of the cells to neutralize HIV-1, we employed a modified version of a TZM-bl neutralization assay adapted from a previous report using NL4-3ΔEnv-GFP pseudotyped with a Clade B envelope, TRO11.^11^^,^^36^ At all cell concentrations tested, only the CD4-GPA expressing cells neutralized the HIV pseudovirus (Figure 3E). While neutralization was only observed at the highest concentration of 6 × 10^7^ cells/mL, the concentration of circulating RBCs in the blood is almost 100-times greater than this (∼5 × 10^9^ cells/mL),^37^ suggesting edited RBCs may serve as effective viral traps *in vivo*. Although the surface area of a K562 cell is much larger than that of an RBC, which may influence CD4 display and viral binding capacity, this comparison nonetheless provides an estimate to allow us to hypothesize which transgene insertion, CD4 alone or CD4-GPA fusion, may serve as more effective viral traps. Overall, these results indicate that only the CD4-GPA cells drove sufficient CD4 receptor expression on the cell surface to facilitate viral entrapment, therefore we chose to proceed in future experiments with only the CD4-GPA constructs.

### Transgene editing preserves native erythroid transcriptional programs

**Figure 4:**
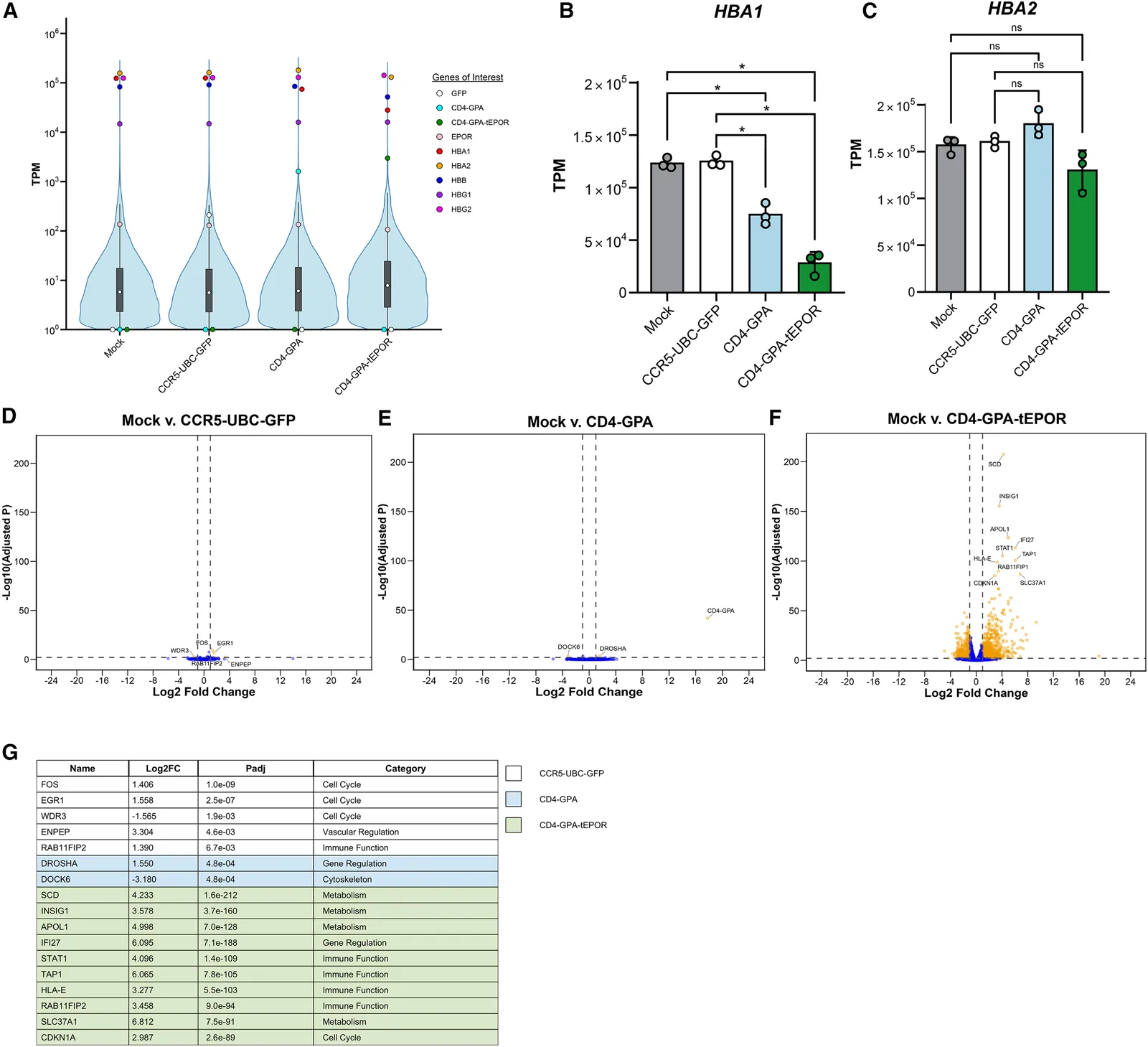
Transcriptomic analysis of transgene-edited, differentiated RBCs (A) RNA-seq transcript abundance in transcripts per million (TPM) for globin genes and transgenes across all conditions. Transgenes and globins are annotated, and values for each gene are averaged across the three biologic donors. (B) TPM values for *HBA1* across all experimental conditions, highlighting differences in expression between edited and unedited samples (*n* = 3, data points and error bars represent mean ± standard deviation). ^∗^*p* = 5.63E-3 for mock vs. CD4-GPA, ^∗^*p* = 1.12E-3 for mock vs. CD4-GPA-tEPOR, ^∗^*p* = 4.77E-3 for CCR5-UBC-GFP vs. CD4-GPA, ^∗^*p* = 9.65E-4 for CCR5-UBC-GFP vs. CD4-GPA-tEPOR by Welch’s two-sample, unpaired, two-tailed *t* tests. (C) TPM values for *HBA2* across all experimental conditions, highlighting differences in expression between edited and unedited samples (*n* = 3, data points and error bars represent mean ± standard deviation). ns, no significance, *p* = 0.088 for mock vs. CD4-GPA, *p* = 0.151 for mock vs. CD4-GPA-tEPOR, *p* = 0.129 for CCR5-UBC-GFP vs. CD4-GPA, *p* = 0.123 for CCR5-UBC-GFP vs. CD4-GPA-tEPOR by Welch’s two-sample, unpaired, two-tailed *t* tests. (D) Volcano plot showing differential gene expression between mock-edited and CCR5-UBC-GFP-edited HSPCs at day 14 of differentiation. Differential expression was assessed using the Wald test. Dashed lines indicate a ±1 log_2_ fold change and an adjusted *p* value threshold of 0.01. Up to 10 of the most significantly up- and down-regulated genes (adjusted *p* ≤ 0.01) are labeled on each plot; all labeled genes are listed in the summary table in (G). (E) Volcano plot showing differential gene expression between mock edited and CCR5-UBC-GFP edited HSPCs at day 14 of differentiation. Analysis as described in (D) (F) Volcano plot showing differential gene expression between mock edited and CCR5-UBC-GFP edited HSPCs at day 14 of differentiation. Analysis as described in (D). (G) Table summarizing the functional categories of the most significantly differentially expressed genes labeled on the volcano plots in (D)–(F). Significance was determined using the parameters defined previously.

To assess whether CD4-GPA or CD4-GPA-tEPOR transgene integration interferes with erythroid differentiation or transcriptional programs, we performed bulk RNA sequencing (RNA-seq) of *in vitro* differentiated genome-edited RBCs. Cord-blood derived CD34^+^ HSPCs were mock edited or edited with CD4-GPA, CD4-GPA-tEPOR, or a genome-editing control (insertion of GFP driven by the constitutive human UBC promoter into the *CCR5* safe harbor locus, hereafter termed CCR5-UBC-GFP) and differentiated into RBCs. Total RNA was isolated from each condition, sequenced, and analyzed for quality and differential expression. Samples yielded an average of 46.5 million reads, with ∼80% aligning to the genome and 99.3% exceeding a quality score of 20 (Figures S5A–S5D). Reads mapped uniquely to all genes and transgenes without overlap. Expression profiles across canonical erythroid markers and associated genes were consistent among all samples, and globin genes were the most abundant transcripts, with *HBA2* and *HBG2* showing the highest expression (Figure 4A). Elevated *HBG2* was consistent with the fetal globin pattern typical of cord blood-derived erythroid cells. Additionally, *HBA1* expression was moderately reduced in *HBA1*-targeted samples, particularly in the CD4-GPA-tEPOR group, reflecting enrichment of edited cells, while *HBA2* expression was similar across all samples (Figures 4B and 4C).

To identify potential transcriptional perturbations, we then compared mock-edited and transgene-edited RBCs for differential gene expression. Notably, only five genes were significantly altered in the CCR5-UBC-GFP-editing control group (Figure 4D). Expression of GFP remained below the threshold to be considered a significantly differentially expressed gene but was exclusively expressed in the CCR5-UBC-GFP condition (Figure S6A). Similarly, only two genes were significantly altered in the CD4-GPA cells compared to mock cells (Figures 4E and S6B), though the magnitude of difference was small. The differentially expressed genes in both groups were primarily involved in general cellular homeostasis and regulatory processes, rather than erythroid-specific pathways (Figure 4G). In contrast, the CD4-GPA-tEPOR group displayed a greater number of transcriptional changes, predominantly affecting genes involved in immune and stress-response pathways (Figures 4F, 4G, and S6C). This pattern is expected, reflecting transcriptional activation downstream of constitutive tEPOR signaling. Importantly, these shifts did not disrupt erythroid maturation, as globin expression (Figure S6D), surface marker acquisition (Figure S6E), and erythroid associated genes were maintained in all groups (Figure S6F). Together, these analyses demonstrate that inclusion of CD4 expressing transgenes at *HBA1* does not induce any global transcriptional dysregulation or disturb expression of genes involved in erythroid maturation.

### Engineered CD4-expressing RBCs retain normal phenotypic maturation, enucleation, and hemoglobin production

**Figure 5:**
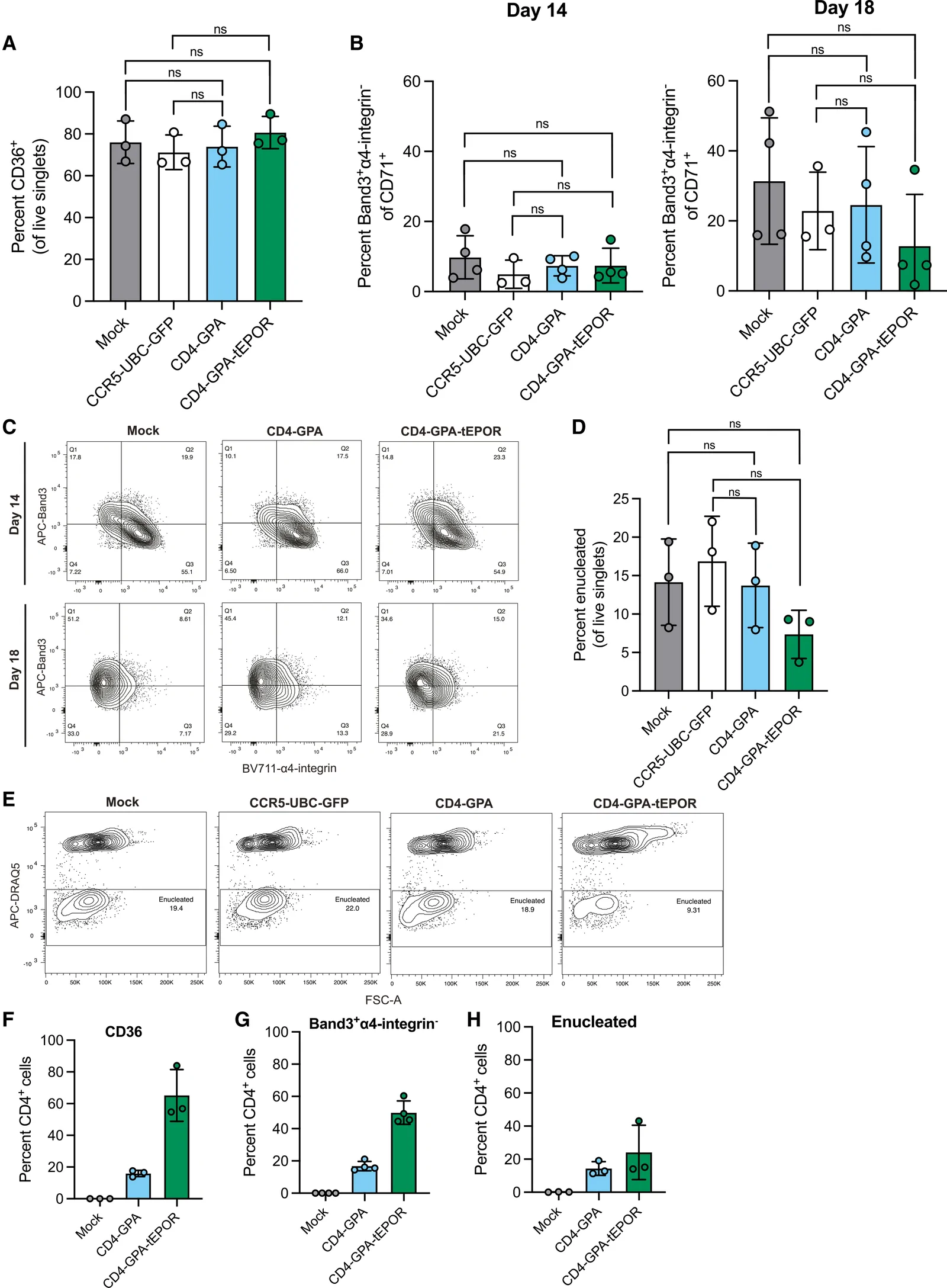
Deeper erythroid phenotypic characterization of genome-edited RBCs (A) Percent CD36^+^ cells of live single cells in each condition on day 14 of RBC differentiation. Bars represent mean ± standard deviation, *n* = 3 biological donors. ns = no significance, *p* = 0.809 for mock vs. CD4-GPA, *p* = 0.566 for mock vs. CD4-GPA-tEPOR, *p* = 0.730 for CCR5-UBC-GFP vs. CD4-GPA, *p* = 0.222 for CCR5-UBC-GFP vs. CD4-GPA-tEPOR by unpaired two-tailed *t* test. (B) Percent Band3^+^ and α4-integrin^-^ cells of live single cells in each condition on day 14 (left) and day 18 (right) of RBC differentiation. Bars represent mean ± standard deviation, *n* = 3 biological donors. ns = no significance, *p* = 0.507 for mock vs. CD4-GPA day 14, *p* = 0.576 for Mock vs. CD4-GPA-tEPOR day 14, *p* = 0.394 for CCR5-UBC-GFP vs. CD4-GPA day 14, *p* = 0.514 for CCR5-UBC-GFP vs. CD4-GPA-tEPOR day 14, *p* = 0.601 for mock vs. CD4-GPA day 18, *p* = 0.163 for mock vs. CD4-GPA-tEPOR day 18, *p* = 0.883 for CCR5-UBC-GFP vs. CD4-GPA day 18, *p* = 0.372 for CCR5-UBC-GFP vs. CD4-GPA-tEPOR day 18 by unpaired two-tailed *t* test. (C) Representative flow cytometry plots of expression of Band3 and α4-integrin in differentiated mock, CCR5-UBC-GFP, CD4-GPA, and CD4-GPA-tEPOR RBCs on day 14 and 18 of RBC differentiation in one donor. Plots shown as percent of live single CD71^+^ cells. (D) Percent enucleated cells as measured by DRAQ5^-^ of live (measured by GhostDye780^-^) single cells in each condition on day 14 of RBC differentiation. Bars represent mean ± standard deviation, *n* = 3 biological donors. ns = no significance, *p* = 0.930 for mock vs. CD4-GPA, *p* = 0.141 for mock vs. CD4-GPA-tEPOR, *p* = 0.535 for CCR5-UBC-GFP vs. CD4-GPA, *p* = 0.068 for CCR5-UBC-GFP vs. CD4-GPA-tEPOR by unpaired two-tailed *t* test. (E) Representative flow cytometry plots showing gating for enucleation in differentiated mock, CCR5-UBC-GFP, CD4-GPA, and CD4-GPA-tEPOR RBCs on day 14. Plots shown as percent of live single cells. (F) Percent CD4^+^ cells of CD36^+^ live single cells on day 14 of RBC differentiation. Bars represent mean ± standard deviation, *n* = 3 biological donors. (G) Percent CD4^+^ cells of Band3^+^α4-integrin^-^ live single cells on day 14 of RBC differentiation. Bars represent mean ± standard deviation, *n* = 3 biological donors. (H) Percent CD4^+^ cells of enucleated live single cells on day 14 of RBC differentiation. Bars represent mean ± standard deviation, *n* = 3 biological donors.

Because gene expression represents only a limited aspect of cell function, we performed deeper phenotypic characterization of normal erythroid development and function of genome-edited RBCs. First, we evaluated several additional markers of RBC development by flow cytometry during *in vitro* differentiation. We found no differences in expression in a marker of early erythroid differentiation, CD36, at day 14 of differentiation (Figure 5A). We then quantified markers of mature erythroid differentiation on both day 14 and day 18 of differentiation. We found that mock, CCR5-UBC-GFP, CD4-GPA, and CD4-GPA-tEPOR-edited cells were all able to gain Band3 expression and lose α4-integrin expression at similar frequencies, as expected for normal RBC differentiation. The Band3^+^α4-integrin^-^ population increased from day 14 to 18 as anticipated as cells were able to mature further (Figures 5B and 5C). Additionally, edited differentiated RBCs were stained for DRAQ5, a marker of nucleated cells, and we found that all groups enucleated at similar frequencies (Figures 5D and 5E). Notably, CD4 expression was maintained in each of the populations throughout RBC maturation, indicating there is no gross selection against CD4-expressing cells during erythroid development (Figures 5F–5H).

To further evaluate whether genome editing impacts the functional properties of RBCs, we assessed RBC deformability in response to oxygen tension using the Laser Optical Rotational Red Cell Analyzer (Lorrca) Oxygenscan system. This assay quantifies how well RBCs elongate under shear stress during deoxygenation and reoxygenation, providing insight into membrane flexibility and cytoskeletal structure. Key parameters include the maximum elongation under full oxygenation (EI_max_) and the minimum elongation during anoxia (EI_min_). Unsurprisingly, we found that overall deformability of the *in vitro* differentiated RBCs was considerably lower than that of adult peripheral blood RBC controls. This is consistent with flow cytometry characterization of *in vitro* derived RBCs showing the majority of cells have not reached full maturation or enucleation. Nonetheless, CD4-GPA and CD4-GPA-tEPOR-edited cells exhibited similar EI_max_ and EI_min_ compared to mock-edited cells, with no evidence of sickling, rigidity, or impaired deformability (Figures S7A and S7B). Additionally, all curves were characteristically flat, indicating the RBCs do not undergo oxygen-dependent rigidity changes. These findings indicate that, as far as our *in vitro* system allows us to investigate, CD4 expression does not disrupt cytoskeletal or membrane integrity or deformability across oxygen levels of RBCs.

Finally, we assessed ability of engineered RBCs to form normal hemoglobin tetramer composition by high-performance liquid chromatography (HPLC). Across all conditions we saw formation of normal adult hemoglobin (HbA) and fetal hemoglobin (HbF) and observed no evidence of aberrant globin switching (e.g., HbC or HbS) (Figures S7C and S7D). Additionally, total hemoglobin, measured by area under the curve (AUC), was comparable or slightly higher in genome-edited cells relative to mock controls, further signifying that neither the *HBA1* editing scheme nor CD4 expression impairs hemoglobin production (Figure S7E). Altogether, these results demonstrate that engineered CD4-expressing RBCs exhibit normal RBC development, deformability, and hemoglobin production, highlighting the safety of our approach.

### HSPC-derived CD4^+^ RBCs neutralize HIV-1

**Figure 6:**
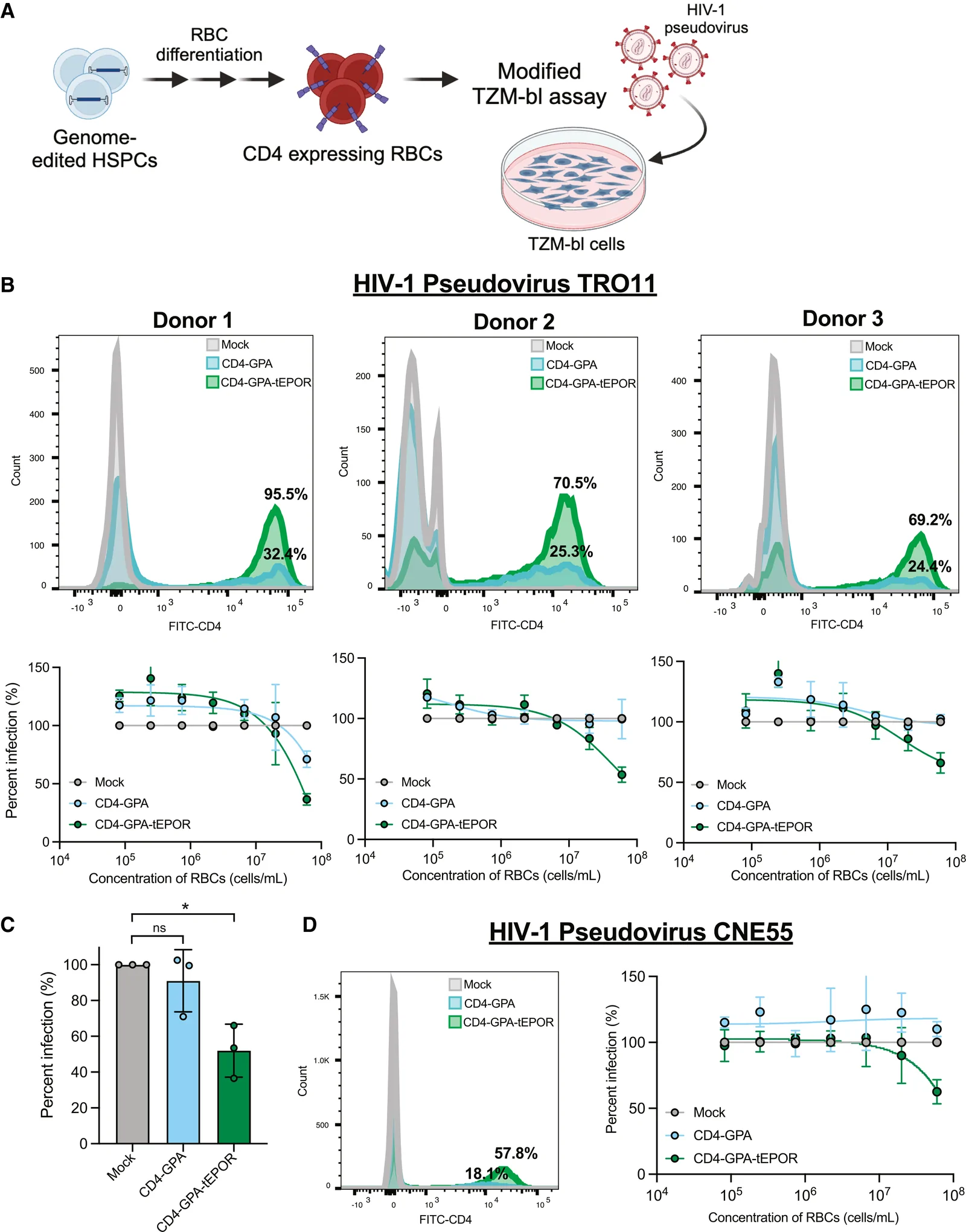
CD4 expressing RBCs neutralize HIV-1 pseudovirus (A) Schematic of genome-editing of HSPCs followed by RBC differentiation and application to the modified TZM-bl assay. (B) Inhibition of infection with TRO11 HIV-1 pseudovirus by differentiated mock or CD4-expressing RBCs of each condition as indicated. Histogram panels (top) show relative CD4 expression of live single cells from each condition and biological donor. Bottom images show percent infection at each concentration of gene-edited RBCs normalized to infection at each concentration of mock edited RBCs. *n* = 3 biological donors shown in separate panels. Data points and error bars represent mean ± standard deviation of technical duplicate infections. (C) Percent infection of TRO11 HIV-1 pseudovirus at the highest cell concentration tested (6 × 10^7^ cells/mL) when normalized to mock edited RBCs. *n* = 3 biological donors also shown in (B). Bars represent mean ± standard deviation of three independent biological donors. ns, *p* = 0.4206 for Mock vs. CD4-GPA, ^∗^*p* = 0.0049 for mock vs. CD4-GPA-tEPOR by unpaired two-tailed *t* test. (D) Inhibition of infection with CNE55 HIV-1 pseudovirus by differentiated mock or CD4-expressing RBCs of each condition as indicated. Histogram panel (right) shows relative CD4 expression of live single cells in each condition. Right images show percent infection at each concentration of cells from genome-engineered RBCs normalized to infection at each concentration of mock-edited RBCs. Values normalized to infection at each concentration of mock edited RBCs. *n* = 1 biological donor. Data points and error bars represent mean ± standard deviation of technical duplicate infections (three mock duplicates were excluded due to mechanical error).

To determine whether HSPC-derived CD4^+^ RBCs were able to neutralize HIV-1, we employed the same editing and erythroid differentiation protocol as aforementioned and then applied the derived RBCs to the modified TZM-bl neutralization assay (Figure 6A). Briefly, CD34^+^ HSPCs were targeted with either CD4-GPA or CD4-GPA-tEPOR followed by RBC differentiation. On day 14, the ability of cells to neutralize HIV-1 was evaluated through a modified TZM-bl assay using TRO11 Env pseudotyped NL4-3. In three assays performed with independent biologic HSPC donors, the CD4-GPA-tEPOR RBCs reduced viral infection at the highest cell concentration tested of 6 × 10^7^ cells/mL (Figure 6B). In contrast, CD4-GPA RBCs only neutralized viral infection in one of the three donors (donor 1) at a concentration of 6 × 10^7^ cells/mL (Figure 6B). None of the lower cell concentrations were able to inhibit infection in cells edited with either construct. On average, CD4-GPA-tEPOR RBCs reached 52% inhibition of infection at the highest cell concentration tested when normalized to mock edited cells, while the less potent CD4-GPA RBCs achieved roughly 10% inhibition of infection (Figure 6C). This difference in the neutralization capacity is likely due to the much greater number of CD4^+^ RBCs generated in the CD4-GPA-tEPOR condition due to enrichment in edited cells driven by the tEPOR (average 27.2% of RBCs in CD4-GPA versus 80.3% in CD4-GPA-tEPOR) (Figures S8A and S8B). Because Clade B envelopes such as TRO11 tend to be easily neutralized, we also tested edited RBC neutralization capacity against a circulating recombinant form (CRF01_AE) envelope, CNE55,^36^ and found similar neutralization capacity as with TRO11 (Figure 6D). This highlights the fact that our strategy is designed to be agnostic to HIV-1 clade or subtype, due to the reliance of all HIV-1 on CD4 binding for infection. Importantly, the highest concentration of CD4-RBCs tested in these experiments (6 × 10^7^ cells/mL) is equivalent to just ∼1.2% of the RBC concentration in human circulation (∼5 × 10^9^ cells/mL).^37^ So although inhibition of HIV-1 in our *in vitro* assays is relatively inefficient, requiring almost 10^8^ RBCs to achieve ∼50% inhibition, the vast abundance of RBCs in circulation supports the potential effectiveness of this approach *in vivo*. Therefore, these results display the potential of CD4-GPA-expressing RBCs to efficiently neutralize virus *in vivo,* even if only a fraction of edited cells engraft and repopulate the blood system.

## Discussion

We present a novel precision genome-editing strategy of HSPCs to produce CD4-expressing RBC viral traps as a treatment for HIV-1 through autologous HSC transplantation (HSCT). There is precedent for using HSCT to treat HIV-1, as the only reported long-term cure of HIV-1 infection is via allogeneic transplantation of *CCR5*-null HSPCs. While this was first reported in 2009 with the Berlin patient,^38^ there have since been several successful uses of *CCR5*-null HSCT in other patients with HIV-1,^39^^,^^40^^,^^41^^,^^42^^,^^43^^,^^44^^,^^45^ highlighting the utility and promise of HSCT for HIV-1 cure. While this is a promising strategy, allogeneic transplantation is not a viable treatment option for most patients due to both the high risks associated with allogeneic transplantation, most notably graft versus host disease (GvHD), and the scarcity of matched donors carrying the *CCR5*-null mutation. Additionally, loss of CCR5 alone is ineffective for patients who carry CXCR4- or dual-tropic HIV-1.^46^^,^^47^ The strategy presented here overcomes each of these barriers. First, engineering and transplanting a patient’s own cells via autologous transplantation removes the need for a matched donor and decreases the risks associated with allogeneic transplantation. Second, engineering HSPCs to express the CD4 receptor on RBC progeny of transplanted cells represents a global strategy which is agnostic to co-receptor tropism or HIV-1 clade, as all HIV-1 strains must use the CD4 receptor to infect cells despite the high global diversity of circulating HIV-1 envelope glycoproteins. Altogether, the work presented here provides a valuable tool as we move toward the development of a one-time strategy for functional cure of HIV-1, while building on the past successes of HSCT.

In this study, we demonstrate that knock-in of CD4 cassettes in HSPCs for RBC-specific expression can produce effective viral traps against HIV-1. While this work builds on previous work using modified RBCs as viral traps against HIV-1,^11^ key innovations presented here include (1) an HSPC-based editing strategy conferring life-long production of therapeutic RBCs, (2) the use of an RNP/AAV6 platform for precise and lineage-specific genome editing, and (3) the incorporation of tEPOR to induce enrichment of CD4-expressing cells. First, we show efficient knock-in of CD4 expression cassettes at the *HBA1* locus in HSPCs using an RNP/AAV6-mediated whole-gene replacement strategy to tie expression to the endogenous *HBA1* promoter (Figure 1). By using the *HBA1* promoter, we restrict the expression of CD4 to only the RBC lineage, minimizing perturbation of other cell lineages and taking advantage of the natural properties of RBCs to serve as viral traps. We then show that when engineered HSPCs differentiate into RBCs *in vitro,* they express CD4 on the cell surface (Figure 2). Aligning with prior work,^11^ we also demonstrate that combining the CD4 with GPA, an abundant RBC cell surface protein, allows for greater expression of CD4 on the cell surface (Figures 2 and 3). Moreover, pairing CD4 expression with that of a truncated erythropoietin receptor (tEPOR) leads to increased production of CD4-RBCs, allowing for approximately 80% of resultant cells to express CD4 by the end of differentiation (Figure 2). Importantly, we demonstrate that engineered RBCs maintain normal erythroid transcriptional programs, phenotypic maturation, and functions (Figures 4 and 5). Lastly, we show that CD4-GPA-tEPOR-edited RBCs can neutralize HIV-1 pseudoviral infection *in vitro*, while CD4-GPA-edited RBCs only showed minimal neutralization in one donor, likely due to the disparity in the number of CD4-expressing cells between conditions (Figure 4).

Because there is roughly a 100-times greater concentration of RBCs in human circulation than we were able to recapitulate *in vitro*, we believe the strategy presented here has the potential to neutralize HIV-1 *in vivo*. Based on the higher expression of CD4 on the cell surface and greater relative abundance of RBCs compared to CD4^+^ T cells, if only 1% of RBCs were derived from engineered HSPCs, there would still be at least 100-times more expression of CD4 on RBCs than on T cells. This would create a hostile environment for HIV that we would expect to lead to the virus being slowly extinguished from the body. These results demonstrate the strong potential of CD4-engineered RBCs to act as viral traps for HIV-1 due to both the greater expression of CD4 on the cell surface and their naturally greater abundance in circulation compared to T cells. In order to further support this hypothesis, we performed rough calculations of the level of engineered HSPC engraftment that may be necessary to achieve neutralizing levels of RBC viral traps in circulation. Considering that CD4-GPA RBCs express roughly ∼3.7× more CD4 than conventional CD4^+^ T cells, we estimate that CD4-GPA RBCs comprising approximately 8% of circulating RBCs would meet the minimum binding neutralization threshold necessary to achieve viral suppression. At this level, CD4^+^ RBCs are predicted to inhibit CD4 binding in the bloodstream by the >99.93% threshold required to suppress viremia to <50 copies/mL in the blood. Because incorporation of tEPOR provides an approximately 3-fold growth advantage during RBC development, we estimate that engraftment as low as 2%–3% edited HSPCs could result in a ∼10% circulating concentration of engineered RBCs (Data S1). Such a low level of engineered cell chimerism could be achievable with non-myeloablative, non-chemotherapy-based conditioning regimens prior to autologous cell transplantation, making this therapy both safer and more accessible for patients. It is important to note that these basic calculations assume a uniform distribution of engineered RBCs and do not account for viral reservoirs outside of the blood or the dynamic exchange of virus between blood and tissue reservoirs. Nevertheless, based on editing and neutralization rates demonstrated in this work, our calculations indicate this strategy has strong potential to be efficacious *in vivo*. However, further non-human primate engraftment and infection challenge experiments will be required to fully evaluate the translational potential of this approach.

While this work provides a promising proof of concept for HSPC-derived RBC viral traps, there are several areas that remain to be explored before this strategy moves toward the clinic. First, we acknowledge that it is now well-known that RNP/AAV6 mediated edited HSPCs exhibit reduced colony formation and decreased engraftment capacity, which is largely attributed to the activation of the DNA damage response and p53 pathway by AAV6.^35^^,^^48^ Despite this, AAV6 remains one of the most efficient delivery methods for donor DNA to HSPCs. While not employed in this work, recent studies have shown that use of small molecules or peptides to inhibit non-homologous end joining (NHEJ) or promote HDR can improve editing efficiency and reduce the amount of AAV6 used, thus enhancing HSPC health.^30^^,^^32^^,^^49^ Alternatively, different DNA donor delivery strategies such as single-stranded oligodeoxynucleotides (ssODNs), lipid nanoparticles, and integrase-deficient lentiviral templates (IDLVs),^31^^,^^50^ are under active investigation and development. As these alternative strategies are improved, the CD4 knock-in described here could be readily adapted for use with a new delivery vector. Furthermore, because it is well-known that immunodeficient mice transplanted with human HSPCs do not reconstitute with many human RBCs in circulation due to phagocytosis by mouse macrophages,^51^^,^^52^ we were not able to evaluate the *in vivo* production of CD4-expressing RBCs in a mouse model. However, previous studies have demonstrated that HSPCs edited to induce HIV-1 resistance are able to facilitate multi-lineage engraftment of human cells *in vivo*.^35^^,^^53^^,^^54^^,^^55^ Future preclinical work will need to define an *in vivo* model to study how long engineered RBCs persist in circulation and how they are cleared. In particular, it will be important to identify whether engineered RBCs are cleared more quickly through the reticuloendothelial system, or if there may be other undesirable clearance pathways of the RBC viral traps, such as through engulfment by macrophages. Additionally, *in vivo* engraftment and infection challenge will be important to understand how effective this RBC-based approach is at reaching and inhibiting infection of certain tissue compartments, such as lymph nodes, that have less blood perfusion, therefore potentially making them less accessible to neutralization by CD4-expressing RBCs. Importantly, engineering RBCs as a viral trap in circulation is complementary to our previously published strategy of arming CD4^+^ T cells and other susceptible hematopoietic cells with intrinsic resistance to CCR5-and CXCR4-tropic HIV-1,^35^ which would provide additional resistance to infection within tissues. Beyond this, *in vivo* models will also allow evaluation of how well RBC viral traps compete with native CD4^+^ T cells as viral targets, an aspect that cannot be readily modeled *in vitro*. Future works may look to HSPC transplantation in the non-human primate (NHP) model for *in vivo* characterization of engraftment of modified cells and subsequent engineered RBC persistence and clearance. In addition, this would allow for investigation of the efficacy of our transplant therapy, as NHPs can be challenged with SHIV.

The use of CD4 viral traps exploits a major vulnerability of HIV-1 because all strains of HIV-1 bind CD4 in canonical infection to enter target cells, thus mutations to escape binding lead to a decrease in viral fitness.^14^^,^^15^^,^^56^^,^^57^ Moreover, because CD4 is naturally found in clusters on the surface of T cells and macrophages,^58^^,^^59^ high expression on the RBC surface will lead to increased physical proximity and avidity to the HIV-1 Env trimer^15^^,^^60^^,^^61^^,^^62^ in order to act as an effective viral trap. By more accurately mimicking the presentation of CD4 on the HIV-1 target cell through presentation on the RBC cell surface, there is decreased likelihood of viral escape compared to that observed when using monomeric forms of CD4.^15^^,^^63^ Despite these advantages, it is well-known that it is important to use multiple means of resistance to combat HIV-1 and the strategy presented here could be readily combined with other CRISPR-Cas9 based strategies being developed by our group and others to engineer HIV-1 resistance, such as *CCR5* KO alone^53^^,^^54^^,^^64^ or in combination with knock-in of either HIV-1 inhibiting proteins^35^ or neutralizing antibodies.^55^ We envision a future where multiple genome-editing strategies could be combined in order to produce HSPCs with potent multilayered resistance against HIV-1, which is essential to combating a virus thought to require at least three separate viral targets of inhibition to prevent treatment escape. In particular, the strategy presented here is especially well positioned to be used in combination with KO or knock-in strategies to *CCR5*, as it functions by knocking into the RBC-specific safe harbor locus *HBA1*, therefore not competing for the same genomic locus. Our group and others have demonstrated feasibility of multiplexed editing at multiple genomic loci in primary cells,^20^^,^^65^^,^^66^ and as this approach improves in the future, engineering resistance at both the *CCR5* and *HBA1* loci has the potential to deliver cells with broader resistance against HIV-1.

Overall, the work presented here showcases a cell-type specific strategy of genome-engineering HSPCs as a potential one-time cure for HIV-1 through harnessing the inherent and highly proliferative capacity of RBCs. Engineering CD4-expressing RBC viral traps builds on promising past work utilizing CD4 as a viral decoy while introducing the advantage of using cell-based carriers. Alongside previous HSPC engineering strategies to intrinsically defend T cells against infection, arm B cells with sustained broadly neutralizing antibodies against HIV-1, and now use RBCs to sequester free virus, genome-edited hematopoietic stem cell transplant therapies provide a unique toolbox for combating complex diseases such as HIV-1. More broadly, this strategy could be easily adapted to engineer expression of other viral receptors to combat a broad spectrum of chronic viral infections.

## Materials and methods

### rAAV6 vector design, production, and purification

CD4 and CD4-GPA sequences were synthesized as gBlock Gene Fragments (Integrated DNA Technologies, IDT, San Jose, CA). Briefly, the CD4 sequence contained the full codon-optimized CD4 sequence while the CD4-GPA sequence was comprised of the CD4 signal peptide and D1D2 domains fused to the N terminus of GPA with a nine amino acid linker.^11^ These fragments were then cloned into an Adeno-associated virus, serotype 6 (AAV6) vector plasmid derived from the pAAV-MCS plasmid (Agilent Technologies, Santa Clara, CA, USA) using restriction enzyme digest or Gibson Assembly (New England Biolabs, Ipswich, MA). rAAV6 vectors were either produced in-house or purchased through SignaGen Laboratories (Frederick, MD). For in-house production, HEK-293 T cells were seeded in ten 15 cm^2^ dishes with 10–12 × 10^6^ cells per plate 24–48 h pre-transfection. Each dish was then transfected with a standard polyethylenimine (PEI) transfection of 6 μg ITR-containing plasmid and 22 μg pDGM6 plasmid (gift from David Russell, University of Washington, Seattle, WA, USA). After 48–72 h incubation, cells were harvested and vectors were purified using the AAVpro purification kit (cat.: 6666; Takara Bio, Kusatsu, Shiga, Japan) as per manufacturer’s instructions. Viral titers were determined using ddPCR to measure the number of vector genomes as previously described.^67^

### CD34^+^ HSPC isolation and culture

Human CD34^+^ HSPCs were isolated from cord blood by the Stanford Binns Program for Cord Blood Research and cultured as previously described.^68^ Samples were obtained with approval from the Stanford Institutional Review Board Committee under protocol 33813. Briefly, isolated mononuclear cells were positively selected for CD34 using the CD34^+^ MicroBead Kit Ultrapure (Miltenyi Biotec, San Diego, CA, cat.: 130-100-453). Cells were cultured at 1.5 × 10^5^–2.5 × 10^5^ cells/mL in CellGenix GMP Stem Cell Growth Medium (SCGM, CellGenix, Freiburg, Germany, cat.: 20802-0500) supplemented with a human cytokine (PeproTech, Rocky Hill, NJ) cocktail: stem cell factor (100 ng/mL), thrombopoietin (100 ng/mL), Fms-like tyrosine kinase 3 ligand (100 ng/mL), interleukin 6 (100 ng/mL), streptomycin (20 mg/mL), and penicillin (20 U/mL), and 35 nM of UM171 (APExBIO, Houston, TX, cat.: A89505). Cells were cultured in a 37°C hypoxic incubator with 5% CO_2_ and 5% O_2_.

### Genome editing of HSPCs

Synthetic chemically modified sgRNAs were purchased from Synthego (Redwood City, CA) or TriLink Biotechnologies (San Diego, CA). Chemical modifications were comprised of 2′-O-methyl-3′-phosphorothioate at the three terminal nucleotides of the 5′ end and the second, third, and fourth bases from the 3′ end as described previously.^69^ The target sequence for the sgRNA targeting *HBA1* was previously published^27^ and is as follows: 5′-GGCAAGAAGCATGGCCACCG-3′. HiFi Cas9 protein was purchased from Aldevron (Fargo, ND, cat:9214). RNPs were complexed at a Cas9:sgRNA molar ratio of 1:2.5 at room temperature for 15–30 min. CD34^+^ cells were resuspended in P3 buffer (Lonza, Basel, Switzerland, cat.: V4XP-3032) with complexed RNPs and electroporated using the Lonza 4D Nucleofector and 4D-Nucleofector X Unit (program DZ-100). Electroporated cells were then plated at 2.5 × 10^5^ cells/mL in the previously described cytokine-supplemented media. Immediately following electroporation, AAV6 was dispensed onto cells at 1.25 × 10^3^–2.5 × 10^3^ vector genomes/cell based on titers determined by ddPCR.

### Generation of K562 stable cell lines

K562 cells (cat: CCL-243, ATCC) were cultured in RPMI 1640 media (cat: 16750-70, VWR) supplemented with 10% bovine growth serum (BGS), and 1% penicillin-streptomycin (RPMI complete). RNPs were complexed the same as aforementioned but using a chemically modified sgRNA targeting CCR5: 5′-TGACATCAATTATTATACAT-3′ that was previously published.^35^ 1 × 10^6^ K562 cells were resuspended in P3 buffer (Lonza, Basel, Switzerland, cat.: V4XP-3032) with complexed RNPs and 250 fmol of donor construct plasmid. Cells were then electroporated using the Lonza 4D Nucleofector and 4D-Nucleofector X Unit (program FF-120) and then replated in 2 mL RPMI complete media. Following recovery and expansion, cells were sorted for GFP using a FACSAria II SORP (BD), twice for purity. Cells were then stained with a CD4-PE (clone: RPA-T4, BioLegend, San Diego, CA, cat: 300508) antibody and analyzed via flow cytometry for confirmation of CD4 expression.

### Allelic modification analysis by ddPCR

Genomic DNA was extracted from cells using QuickExtract DNA extraction solution (Biosearch Technologies, Hoddesdon, UK, cat.: QE09050). To quantify knock-in alleles via ddPCR, we employed *HBA1* specific in-out PCR primers and a probe corresponding to the expected knock-in event (1:3.6 primer to probe ratio). We also used an established genomic DNA reference at the *CCRL2* locus.^70^ The ddPCR reaction was prepared and underwent droplet generation following the manufacturer’s instructions with a Bio-Rad QX200 ddPCR machine (Bio-Rad, Hercules, CA). Thermocycler settings were as follows: 95°C (10 min, 1°C/s ramp), 94°C (30 s, 1°C/s ramp), 57.7°C (30 s, 1°C/s ramp), 72°C (2 min, 1°C/s ramp), return to step 2 for 50 cycles, and 98°C (10 min, 1°C/s ramp). Analysis of droplet samples was then performed using the QX200 Droplet Digital PCR System (Bio-Rad). We divided the copies/μL to determine the frequency of HDR (%): HDR (FAM)/REF (HEX). The following primers and probes were used in the ddPCR reaction.

#### CD4 constructs

Forward Primer (FP): 5′-TGCTGTCCGAGAAGAAAACC-3’

Reverse Primer (RP): 5′-TAGTGGGAACGATGGGGGAT-3’

Probe: 5′-6-FAM/TGCTGGAGTGGGACTTCTCT/3IABkFQ-3’

#### GPA constructs

Forward Primer (FP): 5′-GAAATCGAGAACCCCGAGAC-3’

Reverse Primer (RP): 5′- TAGTGGGAACGATGGGGGAT-3’

Probe: 5′-6-FAM/TGCTGGAGTGGGACTTCTCT/3IABkFQ-3’

#### tEPOR constructs

FP: 5′-TCTGCTGCCAGCTTTGAGTA-3’

RP: 5′-GCTGGAGTGGGACTTCTCTG-3’

Probe: 5′-6-FAM/ACTATCCTGGACCCCAGCTC/3IABkFQ-3’

#### CCRL2 (reference)

FP: 5′- GCTGTATGAATCCAGGTCC-3′

RP: 5′- CCTCCTGGCTGAGAAAAAG -3’

Probe: 5′- HEX/TGTTTCCTC/ZEN/CAGGATAAGGCAGCTGT/3IABkFQ -3’

### INDEL analysis using ICE software

Three days post-editing, QuickExtract solution (Biosearch Technologies, cat.: QE09050) was used to extract genomic DNA from cell samples. The area around the *HBA1* locus cut site was then amplified using the following primer sequences as previously described^30^:

FP: 5′-TGCTTTTTGCGTCCTGGTGTT-3’

RP: 5′-AACGCCTGATCTTGACAGCCC-3’

A 1% agarose gel was used to separate the PCR products, which were then purified using the GeneJETGel Extraction Kit (Thermo Fisher Scientific, cat.: FERK0692). A separate sequencing primer was used for sanger sequencing of the samples: 5′-AGATGGCGCCTTCCTCGC-3’. Sequencing data was analyzed using the ICE CRISPR analysis tool (Synthego, Redwood City, CA, USA) as compared to mock edited control cells.

### Differentiation of HSPCs into erythrocytes

Two to three days following editing of CD34^+^ HSPCs, a 14-day *in vitro* differentiation was performed in supplemented SFEMII medium as previously described.^27^^,^^34^^,^^71^ For the first phase of differentiation, SFEMII base medium was supplemented with 100 U/mL penicillin–streptomycin, 10 ng/mL SCF (PeproTech, Rocky Hill, NJ), 1 ng/mL IL-3 (PeproTech, Rocky Hill, NJ, USA), 3 U/mL EPO (eBioscience, San Diego, CA), 200 μg/mL transferrin (Sigma-Aldrich, St. Louis, MO), 3% human serum (heat-inactivated from Sigma-Aldrich, St. Louis, MO or Thermo Fisher Scientific, Waltham, MA), 2% human plasma (isolated from umbilical cord blood provided by Stanford Binns Cord Blood Program), 10 μg/mL insulin (Sigma-Aldrich, St. Louis, MO), and 3 U/mL heparin (Sigma-Aldrich, St. Louis, MO). Cells were cultured in the first phase of medium for seven days at 1 × 10^5^ cells/mL. In the second phase of medium, days 7–10, cells were maintained at 1 × 10^5^ cells/mL, and IL-3 was removed from the culture. In the third phase of medium, days 11–14, cells were cultured at 1 × 10^6^ cells/mL, with a transferrin increase to 1 mg/mL. When cells were kept past day 14, the third phase of medium was used and cell concentration was adjusted to 5 × 10^6^ cells/mL.

### CFU assay and colony genotyping

Two–three days post-electroporation 500 HSPCs were plated in each well of a SmartDish 6-well plate (STEMCELL Technologies, Vancouver, Canada, cat.: 27370) containing MethoCult H4434 Classic (STEMCELL Technologies, cat.: 04444). After 12–14 days, the wells were imaged using the STEMvision Hematopoietic Colony Counter (STEMCELL Technologies). Colonies were counted and scored with manual correction to determine the number of BFU-E, CFU-GM, and CFU-GEMM colonies. Genomic DNA from individual colonies was extracted using QuickExtract DNA Extraction Solution (Biosearch Technologies, cat.: QE09050). Knockin was then quantified by ddPCR, as described previously, relative to the *TEDDM1* reference gene.

#### TEDDM1 (reference)

FP: 5′-TGGACTATATCAGGCCACAGT-3'

RP: 5′-CAGGAGCCCATCATCAGAATC-3'

Probe: 5′- HEX/TCTCCTGTA/ZEN/TCCTTTGTGCTCTCCCA/3IABkFQ-3’

### Immunophenotyping of differentiated RBCs

Differentiated erythrocytes were analyzed using flow cytometry using a previously published panel of RBC markers.^27^ Cells were stained for 15–30 min at 4°C with the following antibodies: hCD45-V450 (clone HI30, cat: 560367 BD Biosciences, San Jose, CA, USA), CD34-APC (clone 561, cat: 343510 BioLegend, San Diego, CA), CD71-PE-Cy7 (clone OKT9, cat: 25-0719-42, Invitrogen, Santa Clara, CA, USA), CD235a-PE (GPA) (clone GA-R2, cat: 12-9987-82, BD Biosciences), CD4-FITC (clone RPA-T4, cat: 300506, BioLegend), and Ghost Dye Red 780 viability dye (Tonbo Biosciences, San Diego, CA). To analyze more mature RBC markers, cells were first blocked for non-specific binding with human FcR Blocking Reagent (Miltenyi Biotec, cat.: 130-059-901) for 10 min at room temperature and then stained for 20–30 min at 4°C with the following antibody cocktail: CD71-PE-Cy7 (clone OKT9, cat: 25-0719-42, Invitrogen, Santa Clara, CA, USA), and CD235a-PE (GPA) (clone GA-R2, cat: 12-9987-82, BD Biosciences), CD49d-BV711 (α4-integrin) (clone 9F10, cat: 304331, BioLegend, San Diego, CA), CD233-APC (Band3) (clone BRIC 6, cat: 9439, International Blood Group Reference Laboratory, Filton, Bristol, UK), CD4-FITC (clone RPA-T4, cat: 300506, BioLegend), and Ghost Dye Red 780 viability dye (Tonbo Biosciences, San Diego, CA, USA). Samples were analyzed on a FACSAria II SORP (BD). Data was subsequently analyzed using FlowJo (v.10.10.0).

### CD4^+^ T cell isolation and culture

Peripheral blood mononuclear cells (PBMCs) were isolated from LRS chambers obtained from healthy donors using a Ficoll density gradient. Subsequently, CD4^+^ cells were isolated by negative selection using CD4^+^ T cell Isolation Kit, human (Miltenyi, cat: 130-096-533). T cells were cultured at 1 × 10^6^ cells/mL in X-VIVO15 with gentamicin (Lonza), 10% BGS, and 100 IU mL^−1^ human IL-2 (Peprotech) at 37°C with 5% CO2 and ambient O2. For the stimulated condition, cells were activated for 3 days with anti-CD3/anti-CD28 paramagnetic beads (Dynabeads Human T cell Activator, Gibco, cat: 11161D).

### Bulk RNA-seq of differentiated RBCs

Total RNA was extracted and sequenced by Novogene from frozen cell pellets containing 1 × 10^6^ cells per condition. Raw FASTQ files were aligned to the GRCh38 reference genome extended with the relevant transgene sequences. Transcript-level quantification was performed using Salmon, and gene-level counts were summarized with tximport. Differential expression analysis was conducted using DESeq2. Reads with zero counts across all conditions for any donor, as well as mitochondrial transcripts, were excluded. Quality control of sequencing data was performed by Novogene, and all downstream analyses and visualizations were carried out in RStudio.

### RBC deformability measured by Lorrca Oxygenscan assay

Edited erythroid precursor cells (4.5 × 10^7^–6.8 × 10^7^ cells per condition) were spun in an Eppendorf tabletop centrifuge at 500 × *g* for 5 min, resuspended in 1.8 mL of OxyISO (RR Mechatronics) and loaded onto the Laser Optical Rotational Red Cell Analyzer (Lorrca) MaxSis ektacytometer (RR Mechatronics, The Netherlands). Samples were run under the Oxygenscan module to assess edited erythroid precursor deformability, and elongation index was reported.

### Hemoglobin tetramer analysis by HPLC

Frozen cell pellets (1 × 10^6^ cells each) of *in vitro* differentiated RBCs were thawed and lysed in 60–100 μL of HPLC-grade H_2_O for 10 min on ice. Lysates were clarified by centrifugation at 21,000 × *g* for 20 min at 4°C. Hemoglobin was separated and analyzed on a PolyCAT A cation-exchange column (100 × 2.1 mm; 3 μm, 1,500 Å; PolyLC, Inc.) using an Agilent 1260 Infinity II HPLC system equipped with a diode array detector set to measure at 415 nm. Mobile phase A consisted of 40 mM Bis-Tris and 3 mM KCN in HPLC-grade H2O, adjusted to pH 6.5 with HCl. Mobile phase B was prepared similarly with the addition of 200 mM NaCl and adjusted to pH 6.8. Hemoglobin retention times were verified using Variant Hemoglobin Control, AFSC (Analytical Control Systems, Inc.) and relative hemoglobin expression was reported as ratio comparisons.

### Modified TZM-bl neutralization assay

The modified TZM-bl HIV-1 pseudotype neutralization assay was adapted from a previously described protocol.^11^ TZM-bl cells were obtained through the NIH AIDS Reagent Program (cat.: 8129) and cultured in DMEM with 10% BGS, and 1% penicillin-streptomycin (complete DMEM) at 37°C, 5% CO2, and ambient oxygen levels. HIV-1 env pseudotyped lentivirus was produced as previously described using a pNL4-3ΔEnvGFP plasmid and global *env* panel plasmids provided by the NIH AIDS Reagents Program (cat: 11100 and cat: 12670, respectively).^36^^,^^72^ Differentiated erythrocytes were resuspended in complete DMEM with 10 μg/mL polybrene (cat: TR1003G, Fisher Scientific), serial dilutions were performed, and dilutions were moved to a 48 well plate. HIV-1 pseudovirus was then added to each well and plate was incubated for 4 h while shaking at 600 rpm at room temperature. 1 × 10^4^ TZM-bl cells were plated in black-walled, clear-bottom 96-well plates (Corning, Corning, NY, cat.: 07-200-565). RBCs were then spun down at 500 × g for 10 min in a 96-well U-bottom plate (in duplicate) and supernatant was then moved to the well of the black-walled, clear-bottom 96-well plates containing TZM-bl cells. Cells were incubated for 48 h then measured for luciferase signal corresponding to infection. Briefly, cells were lysed with Reporter Lysis Buffer (Promega, Madison, WI, cat.: E3971) and freeze-thawed at −80°C. Lysed samples were read for relative luminescence units (RLU) using a Synergy H1 plate reader (BioTek, Winooski, VT) that injected luciferin solution consisting of the following: 200 mM Tris [pH 8], 10 mM MgCl_2_, 300 μM ATP, Firefly Luciferase Signal Enhancer (Thermo Fischer Scientific, cat. 16180), and 150 μg/mL d-luciferin (Biosynth Chemistry & Biology, Staad, Switzerland, cat.: L8220). Percent infection was determined by normalizing RLU values to the RLU of mock-edited RBC wells using GraphPad Prism v.10.

### Statistical analysis

GraphPad Prism v.10 software was used for all statistical analysis.

## Data and code availability

The main data supporting the results in this study are available within the article and its supplementary figures. Sequencing data generated for RNA sequencing analysis have been deposite in the NCBI Gene Expression Omnibus (GEO)^73^ and are accessible through GEO: GSE338343. Sequences of the sgRNA and ddPCR primers and probes are provided in materials and methods section. All data generated in this study are available from the corresponding authors upon reasonable request.

## Acknowledgments

We thank the NIH AIDS Reagent Repository for providing the pNL4-3ΔEnvGFP and Global *env* pseudotyping panel plasmids for the HIV-1 infection experiments. We thank the Stanford Institute for Stem Cell Biology and Regenerative Medicine FACS Core for their support and access to the flow cytometry machines. We thank the Binns Program for Cord Blood Research for providing purified cord blood HSPCs. S.E.L. was supported by the Stanford Medical Scholars Research Program, the American Society of Hematology Minority Medical Student Award Program, the Stanford Medical Scientist Training Program, and NIH F30 Individual Predoctoral Fellowship (1F30HL178496-01). W.N.F. was supported by the Stanford T32 Graduate Training Program in Stem Cell Biology and Regenerative Medicine (5T32GM119995) and the Blavatnik Family Foundation as a Blavatnik Fellow. F.K.E. was supported by the Stanford Knight-Hennessy Scholarship, Paul And Daisy Soros Fellowship for New Americans, and the Hertz Fellowship. A.M.D. was supported by NIH F32 Individual Postdoctoral Fellowship (1F32HL154667-01), NIH K99/R00 Pathway to Independence Award (1K99HL172253-01), HARC Center Collaborative Developmental Award 2024-2026 (Parent U54 AI170792), and the Carver College of Medicine Department of Microbiology Endowed Scholar Fund. This work was partially supported by the University of Iowa Year 2 P3 Strategic Initiatives Program through funding received for the project entitled “High Impact Hiring Initiative (HIHI): A Program to Strategically Recruit and Retain Talented Faculty.” M.H.P. was supported by the Sutardja Chuk Professorship in Definitive and Curative Medicine and the Laurie Kraus Lacob Translational Medicine endowment.

## Author contributions

S.E.L, W.N.F., K.B.-E, A.P., F.K.E., M.M-G., B.H-N, K.R., Z.K., N.A.A., N.M.J., H.Y.G., and A.M.D. contributed to experimental design, performance, and analysis. S.E.L, W.N.F., K.B.-E., and M.H.P. wrote the draft and finalized versions of the manuscript with input from other authors. A.M.D and M.H.P. supervised the project.

## Declaration of interests

M.H.P. serves on the scientific advisory board of Allogene Tx and is an advisor to Versant Ventures. M.H.P. has equity in CRISPR Tx and has equity and is a founder of Kamau Therapeutics.

## Supplemental information

Document S1. Figures S1–S8

Data S1. Mathematical Calculations for CD4-RBC Mediated Neutralization

Document S2. Article plus supplemental information
