# Supplementary material for: Engineering hematopoietic stem and progenitor cells to generate red blood cells as viral traps against HIV-1
Source: Mol Ther Adv. 2026 Jun 29;34(3):201799. doi: 10.1016/j.omta.2026.201799 (PMC13519646; doi:10.1016/j.omta.2026.201799)
Supplement: Supplementary file 1 [file mmc1.pdf]

OMTA, Volume *34*

Supplemental information

Engineering hematopoietic stem and progenitor cells to generate red blood cells as viral traps against HIV-1

Sofia E. Luna, William N. Feist, Kaya Ben-Efraim, Alex Pendergast, Freja K. Ekman, Britney Hernandez-Naughton, Megan Merritt-Garza, Kathleen Romero, Zak Kostamo, Nelson A. Amorin, Nicole M. Johnston, Hana Y. Ghanim, Vivien Sheehan, Amanda M. Dudek, and Matthew H. Porteus

Mathematical Calculations for CD4-RBC Mediated Neutralization

(1) Viral load context in chronic HIV infection

- Average viral load in chronic untreated HIV infection: ~75,000 copies/mL of plasma[1,2]
- Since each virion contains two RNA copies: ~ 37,500 virions/mL
- With an average plasma volume of 3,500 mL[3], the total plasma viral load is:

$$V_{initial} = 37,500 \cdot 3,500 = 1.3125 \cdot 10^8 \textit{ virions}$$

Viral suppression is clinically defined as < 50 RNA copies/mL[4], equivalent to 25 virions/mL. Across 3,500 mL of plasma, this target corresponds to:

$$V_{target} = 25 \cdot 3,500 = 8.75 \cdot 10^4 \textit{ virions}$$

(2) Required viral clearance

$$\Delta V = V_{initial} - V_{target} = 1.3125 \cdot 10^8 - 8.75 \cdot 10^4 = 1.311625 \cdot 10^8$$

$$\% \textit{ removed} = \frac{\Delta V}{V_{initial}} \cdot 100 = \frac{1.311625 \cdot 10^8}{1.3125 \cdot 10^8} \approx 99.93\%$$

The required viral clearance to achieve clinical suppression is ~99.93%.

(3) Determining Relevant Cell Numbers

- Total RBCs (R) in an average healthy adult[5]:

$$R = 5 \cdot 10^6 \frac{RBCs}{uL}$$

- Total CD4+ T cells ($T_{Healthy}$) in an average healthy adult:[6]

$$T_{healthy} = 1 \cdot 10^3 \ \frac{CD4^+ \ T\ cells}{uL}$$

We first determined the average number of RBCs and $CD4^+$ T cells in an average healthy individual. Since our therapy neutralizes HIV by binding free virions in the plasma before they can attach to target cells, the relevant target-cell population includes all circulating $CD4^+$ T cells.

(4) Calculation of RBC:activated CD4+ T-cell Ratio and Relative Binding Potential

- Starting ratio ($R{:}T_{HIV}$)

$$5 \cdot 10^6 \ RBCs: 1 \cdot 10^3 \ CD4^+ \ T\ Cells \ \rightarrow 5\,000 : 1$$

- Relative binding coefficient:
  - K_T = 1 (baseline binding capacity of a single $CD4^+$ T cell, normalized)
  - K_R = 3.7 (RBC binding coefficient, based on RBC CD4 median fluorescence intensity (MFI) vs. Stimulated $CD4^+$ T cells)

K_R was calculated from the fold-change in MFI of $CD4^+$ RBCs relative to stimulated $CD4^+$ T cells. Activated $CD4^+$ T cells are the most susceptible to HIV infection. While approximately 22% of $CD4^+$ T cells consist of stimulated cells during chronic HIV infection (as reported in prior studies), our calculations assume 100% stimulation as a more cautious estimate.[7]

- Effective CD4-RBC binding capacity against HIV was then calculated as:

$$R_{eff} = R \cdot \frac{K_R}{K_T} = 5\,,000 \ \cdot 3.7 = 18\,,500 \ \frac{CD4^+ \ binding\ units}{uL}$$

This indicates that, if every circulating RBC expressed CD4, the total RBC population would be equivalent to roughly 18,500 activated $CD4^+$ T cells per μL in terms of HIV binding capacity.

(5) Minimum Estimate of RBCs for Viral Neutralization

To determine the minimum fraction of circulating RBCs required for binding suppression, we first establish that:

$$F_{neutralized} = \frac{R_{eff}}{R_{eff} + T}$$

and

$$R_{eff,\, min} = F_{RBC,\, min} \cdot R_{eff}$$

Plugging into the equation below, we can now solve for $F_{RBC,\, min}$:

$$F_{neutralized,\, min} = \frac{F_{RBC,\, min} \cdot R_{eff}}{F_{RBC,\, min} \cdot R_{eff} + T}$$

Where:

- $F_{neutralized,\, min.}$ = 0.9993 (99.93%)
- T = 1 $CD4^{+}$ binding unit/μL, normalized
- R_{eff} = 18,500 $CD4^{+}$-equivalent binding units/μL

This suggests that only ~7.7% of circulating RBCs need to carry the edit to meet the minimum binding neutralization threshold.

(6) HSC Engraftment Required to Achieve $F_{RBC,\, min}$

Based on our data, cells carrying tEPOR are expected to increase ~3-fold growth advantage, only 2.56% of edited HSCs (x) would need to engraft, compared with 7.7% without tEPOR.

Based on our data demonstrating the average knock-in (KI) efficiency is approximately 25%, we can solve for the percentage of total HSCs that would need to engraft.

$$F_{engrafted\, HSC} = \frac{x}{KI} = \frac{0.0256}{0.25} = 0.1024$$

With the tEPOR advantage, ~10% of the total transplanted HSCs and fewer than 3% of edited HSCs would need to engraft to reach the minimum $CD4^{+}$ RBC level (~8%) predicted to inhibit HIV binding in the blood stream by more than 99%. Conversely, 30.8% of total transplanted HSCs would need to engraft in the absence of tEPOR.

References

1. Piatak M Jr et al. High levels of HIV-1 in plasma during all stages of infection determined by competitive PCR. *Science*. 1993;259(5102):1749-1754. doi:10.1126/science.809608
2. Sterling TR et al. Initial plasma HIV-1 RNA levels and progression to AIDS in women and men. *N Engl J Med*. 2001;344(10):720-725. doi:10.1056/NEJM200103083441003
3. Mallick, Abhiram, and Andrew R. Bodenham. “Regulation of Blood Volume and Electrolytes.” Foundations of Anesthesia, 2nd edition, 2006. ScienceDirect, doi:10.1016/B978-0-12-813257-5.01107-X.
4. Hill A, Cooper DA. Should we now adopt the HIV-RNA <50 copy endpoint for clinical trials of antiretroviral-experienced as well as naïve patients? *AIDS*. 2007;21(12):1535-1539. doi:10.1097/QAD.0b013e3281f59f7e
5. Mayo Clinic Staff. High red blood cell count. *Mayo Clin*. Accessed 2025. https://www.mayoclinic.org/symptoms/high-red-blood-cell-count/basics/definition/sym-20050858
6. Cleveland Clinic. CD4 cell count (T-cell test). *Cleveland Clinic*. Accessed 2025. https://my.clevelandclinic.org/health/diagnostics/cd4-count
7. Kestens, L et al. “Selective increase of activation antigens HLA-DR and CD38 on CD4+ CD45RO+ T lymphocytes during HIV-1 infection.” *Clinical and experimental immunology* vol. 95,3 (1994): 436-41. doi:10.1111/j.1365-2249.1994.tb07015.x
8. Davenport MP et al. Functional cure of HIV: the scale of the challenge. *Nat Rev Immunol* 19, 45–54 (2019). https://doi-org.stanford.idm.oclc.org/10.1038/s41577-018-0085-4
